# Mechanistic and Preliminary Safety Profiling of a Multicomponent Natural Product-Based Injectable Formulation Targeting Skin Aging-Related Pathways: A Network Pharmacology and Single-Dose Toxicity Study

**DOI:** 10.3390/ph19081317

**Published:** 2026-08-20

**Authors:** Ji Hye Hwang, Chul Jung

**Affiliations:** 1 Department of Acupuncture & Moxibustion Medicine, College of Korean Medicine, Gachon University, Seongnam 13120, Republic of Korea; 2 Namsangcheon Korean Medicine Clinic, Seoul 06656, Republic of Korea; jcnu2000@hanmail.net

**Keywords:** network pharmacology, skin aging, natural products, injectable formulation, pharmacopuncture, estrogen signaling, cellular senescence, oxidative stress, subcutaneous toxicity

## Abstract

**Background/Objectives:** Skin aging involves interconnected inflammatory, oxidative, hormonal, extracellular matrix (ECM), and cellular senescence-related mechanisms, supporting the need for multitarget approaches. This study aimed to evaluate the effects of a multicomponent natural product-based injectable formulation developed in Korean medicine practice, Dong-An Pharmacopuncture (DAP), on skin aging-related pathways. **Methods:** A network pharmacology approach was used to identify the active compounds, predicted molecular targets, and signaling pathways associated with DAP. Sixty-two active compounds from 11 constituent materials were screened, and 70 final targets were identified using STITCH-based prediction, intersection with GeneCards-derived skin aging-related targets, and quality filtering. **Results:** Herb-compound-target network analysis yielded 225 compound–target interactions across 292 edges. Protein-protein interaction analysis identified a highly connected network with 1,334 edges, and hub analysis converged on 10 core targets: *TNF*, *IL6*, *ESR1*, *TP53*, *AKT1*, *PPARG*, *EGFR*, *PTGS2*, *CASP3*, and *PPARA*. Gene Ontology and Kyoto Encyclopedia of Genes and Genomes enrichment analyses identified four major mechanistic axes: inflammatory and oxidative stress regulation, hormonal skin homeostasis, tissue repair and ECM remodeling, and cellular senescence-related regulation. Because DAP is administered by injection, a Good Laboratory Practice-compliant single-dose subcutaneous toxicity study was additionally conducted in Sprague–Dawley rats, which showed no mortality, abnormal clinical signs, or histopathological findings attributable to DAP at 1.0 mL/head. **Conclusions:** The findings in this study provide a systems-level framework for the predicted multitarget mechanisms of DAP in skin aging-related pathways and support the need for further experimental validation of its predicted mechanisms and repeated-dose safety.

## 1. Introduction

Skin aging is a complex biological process driven by oxidative stress, chronic low-grade inflammation, hormonal changes, extracellular matrix (ECM) remodeling, and cellular senescence. Ultraviolet (UV) radiation and environmental pollutants generate reactive oxygen species (ROS) that activate NF-κB-mediated inflammatory responses, increase matrix metalloproteinase (MMP) activity, and impair collagen homeostasis, thereby contributing to dermal degeneration [1,2]. Hormonal decline, particularly reduced estrogen signaling in perimenopausal and postmenopausal women, may further affect skin barrier function, elasticity, and collagen metabolism [3,4]. Oxidative stress-induced DNA damage and cellular senescence can promote the senescence-associated secretory phenotype, thereby maintaining a proinflammatory dermal microenvironment [5]. These interconnected mechanisms suggest that skin aging may be better addressed through multitarget strategies rather than the modulation of a single pathway [6].

Natural products and multicomponent formulations have attracted interest as potential candidates for skin aging-related conditions because they contain diverse bioactive constituents capable of interacting with multiple molecular pathways [7,8]. Compounds such as polyphenols, flavonoids, terpenoids, alkaloids, saponins, and steroid-like molecules are associated with antioxidant defense, inflammatory regulation, hormonal signaling, and ECM remodeling [9,10]. However, the complexity of these formulations makes it difficult to characterize their integrated mechanisms using conventional single-target pharmacological approaches. Network pharmacology, which integrates compound–target–pathway relationships at the system level, can be used as a hypothesis-generating tool to organize predicted compound–target relationships and prioritize putative pathways for further experimental validation [11,12].

Pharmacopuncture is an injectable treatment modality used in Korean medicine, in which herbal or natural product-derived preparations are administered to acupoints or target tissue sites. For cutaneous applications, this delivery route may be relevant because it provides a non-oral mode of administration and allows investigation of local tissue exposure to poorly absorbed constituents [13,14]. Nevertheless, as pharmacopuncture formulations are administered by injection, both mechanistic plausibility and route-relevant safety evaluations are important prior to further translational development.

Dong-An Pharmacopuncture (DAP) is a multicomponent, natural product-based injectable formulation developed in contemporary Korean medicine practice. The term “dong-an” denotes a youthful facial appearance in Korean and is used here as the formulation name. DAP consists of an eight-component base formulation, as previously described in pharmacopuncture research, together with three additional skin-oriented components: *Centella asiatica*, Hominis Placenta, and *Panax quinquefolius*. This triad of components has been individually associated with collagen synthesis, wound healing, MMP regulation, hormonal or regenerative signaling, and antioxidant or anti-inflammatory pathways [15,16,17,18,19,20]. DAP represents a contemporary injectable formulation built on an established pharmacopuncture base and modified by adding skin-oriented constituent materials. Although the base formulation has previously been analyzed using a network pharmacology approach in a liver injury-related context [21], these findings do not directly establish the dermatological relevance of the modified eleven-component formulation. Therefore, the integrated compound–target–pathway network of DAP in relation to skin aging remains to be systematically characterized.

This study aimed to evaluate the predicted mechanistic profile and preliminary route-relevant safety of DAP as a multicomponent natural product-based injectable formulation in relation to skin aging-related pathways. We hypothesized that DAP would exhibit a multi-target network distribution involving inflammatory, oxidative stress-related, hormonal, ECM remodeling, and cellular senescence-related pathways relevant to skin aging, and that single-dose subcutaneous administration would not produce overt acute toxicity under the tested conditions. To test this, we applied a network pharmacology workflow to identify active compounds, predicted molecular targets, herb-compound-target (H-C-T) relationships, hub targets, and enriched biological pathways. In addition, because DAP is administered via subcutaneous injection, we conducted a Good Laboratory Practice (GLP)-compliant single-dose toxicity study in rats to provide preliminary route-relevant safety information. The findings provide a systems-level mechanistic framework for the predicted multi-axis profile of DAP and preliminary nonclinical safety information supporting further experimental validation.

## 2. Results

### 2.1. Active Compound Collection and Target Identification

A total of 62 active compounds were identified from the 11 constituent materials of DAP (Table 1) through integrated database mining using the Traditional Chinese Medicine Systems Pharmacology (TCMSP) and HERB, together with literature-based curation (Table S1). The compound set included bioactive constituents derived from an eight-component formulation and three additional skin-oriented components. The base formulation contained major compounds such as muscone, berberine, palmatine, baicalin, baicalein, wogonin, oxymatrine, matrine, costunolide, dehydrocostus lactone, oleanolic acid, anemonin, and bile acid-related derivatives. The three additional skin-oriented components contributed ginsenosides from *P. quinquefolius*; steroid hormones and melatonin from Hominis Placenta; and asiaticoside, madecassoside, quercetin, apigenin, kaempferol, ursolic acid, and ascorbic acid from *C. asiatica*. As a supplementary marker-based chemical confirmation, high-performance liquid chromatography–ultraviolet (HPLC-UV) analysis confirmed the presence of asiaticoside in the DAP batch used in the toxicity study (lot no. NN0726001) with a measured content of 0.351 mg/vial, meeting the predefined specifications (Table S2).

Compound–target interactions were predicted using the Search Tool for Interactions of Chemical (STITCH) database (combined confidence score ≥ 0.4), initially yielding 103 unique protein targets. Skin aging-related disease targets were independently collected from GeneCards using seven distinct search terms: “skin anti-aging,” “skin elasticity,” “facial aging,” “cellular senescence,” “cellular aging,” “tissue regeneration,” and “wound healing,” generating 10,015 disease-related entries. An intersection analysis between the compound-predicted targets and skin aging-related disease targets identified 77 candidate targets. An additional eight targets with strong literature-based compound–target evidence not captured by STITCH were manually supplemented: *COL1A1* [15], *TGFB1* [15], *NFE2L2* [7], *AKT1*, *PIK3CA* [22], *TYR* [20,23], *TNF*, and *IL6* [10,24,25], resulting in a preliminary set of 85 targets.

**Figure 1:**
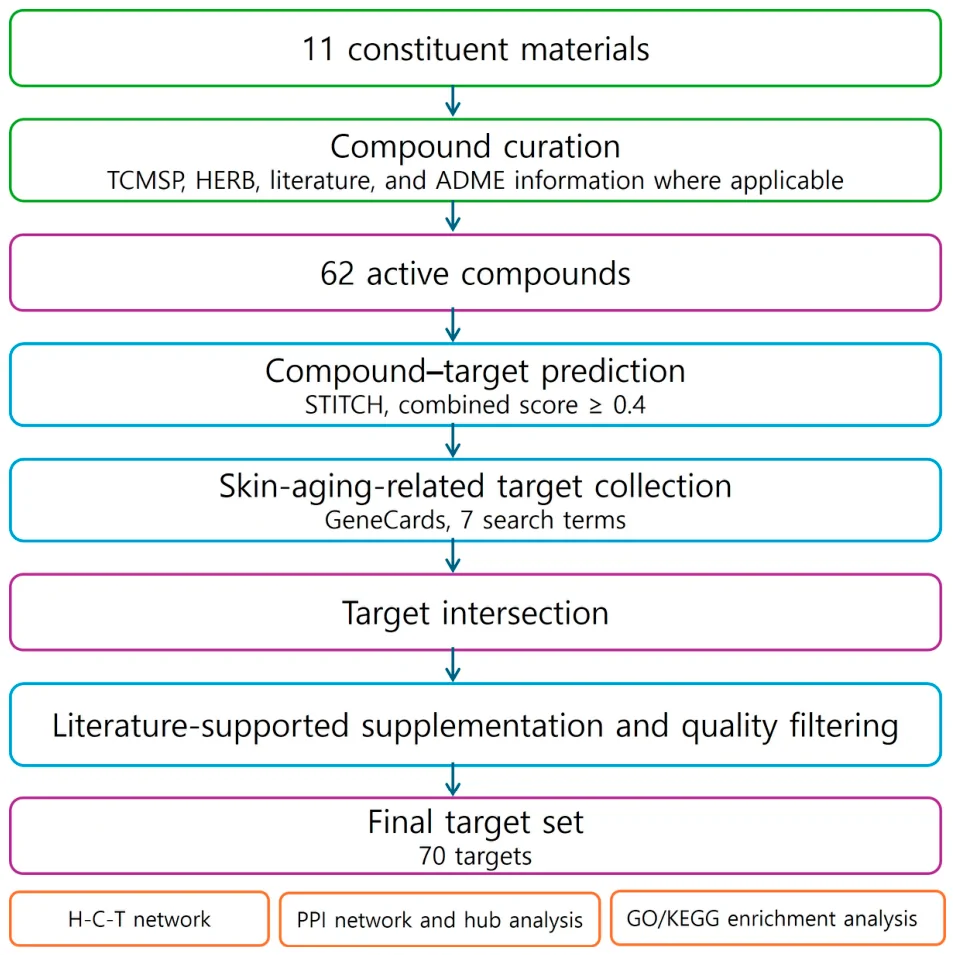
Workflow of compound curation, target identification, quality filtering, and downstream network pharmacology analysis of DAP. Compounds from the 11 constituent materials were curated using TCMSP, HERB, literature sources, and ADME information where applicable. Compound-related targets were predicted using STITCH and intersected with skin-aging-related targets collected from GeneCards. Literature-supported supplementation and quality filtering were then performed to generate the final target set for H-C-T network construction, PPI network and hub analysis, and GO/KEGG enrichment analysis.

Quality filtering was applied to exclude targets in the following categories: (1) RNA polymerase II subunits (*POLR2A*, *POLR2B*, *POLR2C*, *POLR2E*, *POLR2G*, and *POLR2H*), *INTS10*, and *SUPT5H*, which lacked compound-level interaction evidence in the STITCH database and are not conventionally used as pharmacological targets; (2) *GAPDH* and *GAPDHS*, excluded as ubiquitous housekeeping genes with non-specific interaction profiles; (3) targets with low predicted relevance to skin aging-related pathways (*HMOX2*, *NPPA*, *NPPB*, *REN*, *UGT1A4*, and *UGT1A6*); (4) protein–protein interaction (PPI)-isolated nodes (*VEGFA*, *LDHB*, and *ME2*) showing no edges in the STRING PPI network at the applied confidence threshold. Three biologically relevant targets, *HMOX1* [7], *PTGS2* [10,25], and *MMP13* [16], were retained based on STITCH or literature-based evidence, yielding a final target set of 70 genes utilized in all downstream networks and enrichment analyses. Target-mapped active compounds dictated network construction, whereas the broader candidate compound set identified during compound curation was reserved for absorption, distribution, metabolism, and excretion (ADME) profiling as a supplementary analysis of the formulation’s overall physicochemical profile. The overall workflow from compound curation to downstream network pharmacology analysis is summarized in Figure 1.

### 2.2. H-C-T Network Analysis

**Figure 2:**
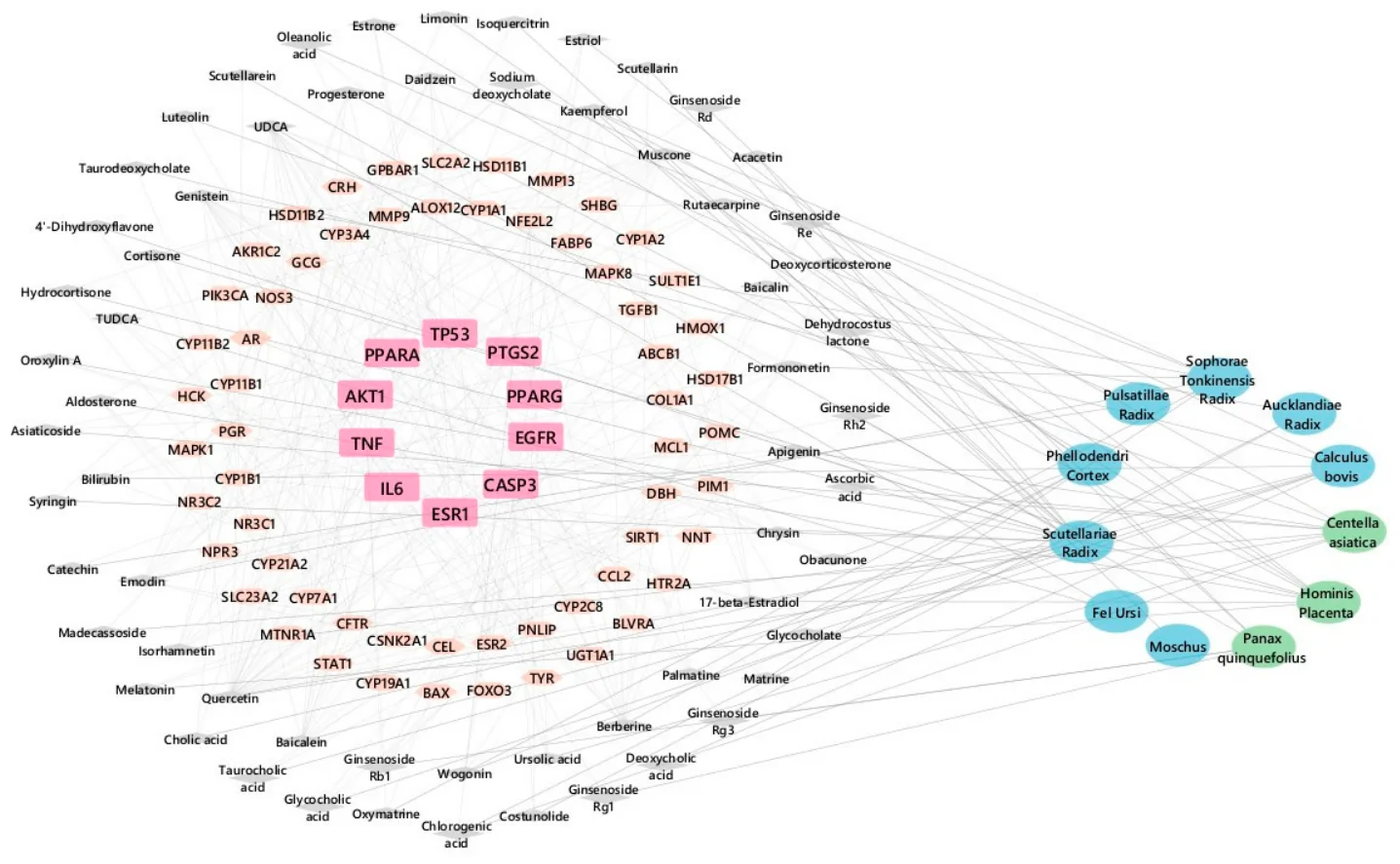
Herb-compound-target network of DAP. Nodes represent constituent materials (n = 11), active compounds (n = 62), and predicted targets (n = 70). Edges represent herb–compound (n = 67) and compound-target interactions (n = 225). Hub targets are highlighted in pink.

The H-C-T network was constructed using Cytoscape and comprised 11 constituent material nodes, 62 compound nodes, and 70 target nodes connected by 225 compound–target edges across 292 total edges, including herb-compound connections (Figure 2). Compound–target interactions were classified according to evidence sources, including STITCH-based predictions, literature-supported interactions, and combined STITCH–literature evidence.

Among the constituent materials, *Scutellariae radix* exhibited the highest compound connectivity, followed by Hominis Placenta and *C. asiatica*. At the compound level, quercetin showed the highest degree, followed by berberine and baicalein. Multicomponent convergence analysis revealed that *NFE2L2* received input from the largest number of constituent materials, followed by *CASP3* and *AKT1*. These findings suggest that DAP-related compounds converge on several shared molecular nodes associated with oxidative stress regulation, apoptosis, and cell survival. However, these network-level observations should be interpreted as hypothesis-generating rather than as evidence of additive or synergistic pharmacological effects.

A component-group-level summary of the predicted targets is presented in Table S3. This summary serves solely to describe the distribution of predicted targets within DAP, without inferring additive, synergistic, or comparative efficacy.

### 2.3. PPI Network Analysis and Hub Target Identification

**Figure 3:**
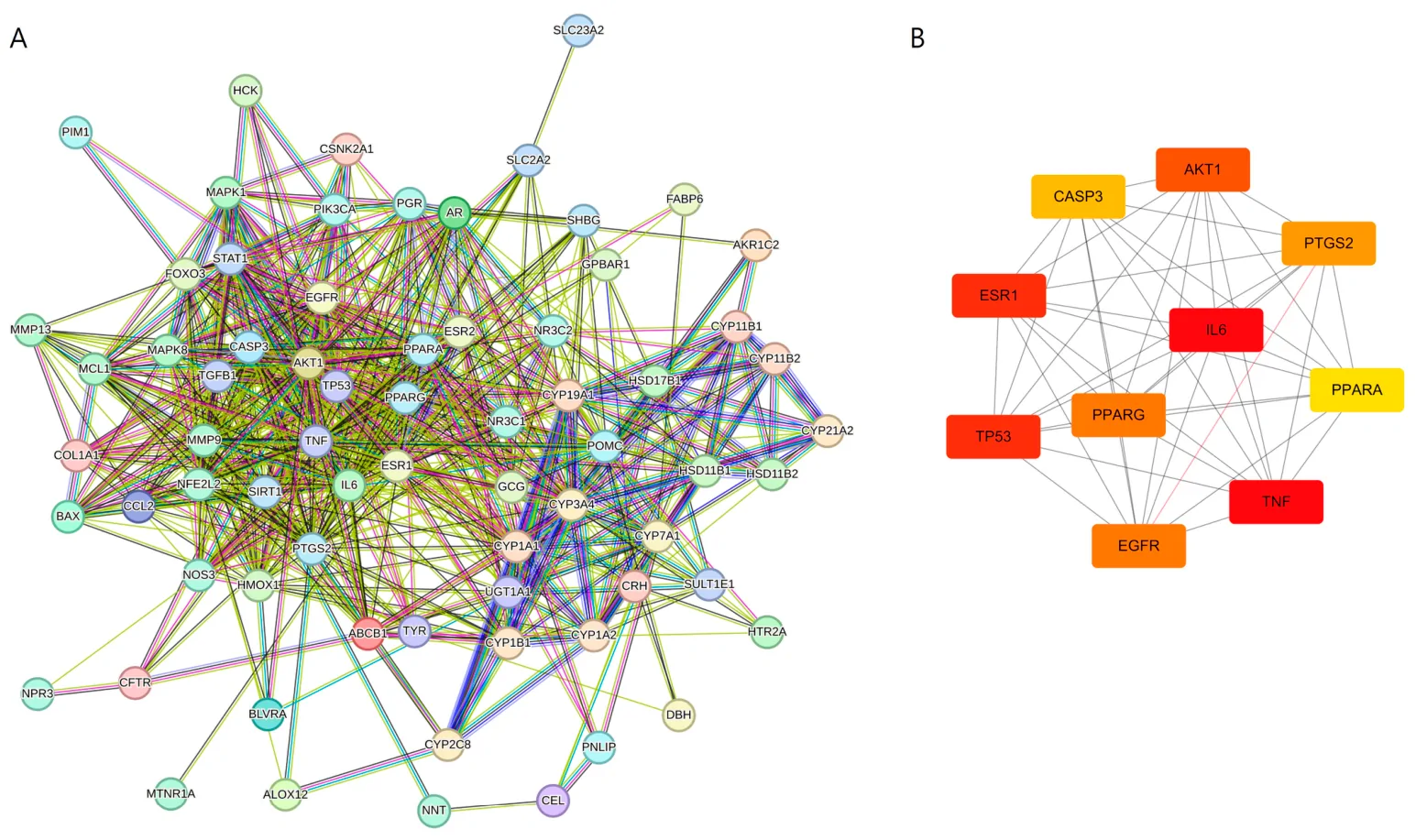
Protein-protein interaction network and hub target analysis of predicted DAP targets. (**A**) Protein–protein interaction network of 70 final targets constructed using STRING with a confidence threshold ≥ 0.4. (**B**) Core hub target network showing the top 10 targets ranked by degree centrality: *TNF*, *IL6*, *TP53*, *ESR1*, *AKT1*, *PPARG*, *EGFR*, *PTGS2*, *CASP3*, and *PPARA*.

The PPI network was constructed using STRING with a confidence threshold ≥ 0.4. The final network contained 70 nodes and 1334 edges, with a network density of 0.276, clustering coefficient of 0.659, and characteristic path length of 1.892 (Figure 3). Hub targets, including degree, betweenness, and closeness centralities, were identified using CytoHubba and NetworkAnalyzer.

Ten targets that ranked highest by degree centrality were defined as core hub targets: *TNF*, *IL6*, *TP53*, *ESR1*, *AKT1*, *PPARG*, *EGFR*, *PTGS2*, *CASP3*, and *PPARA*. MCC analysis highlighted *SIRT1* and *NFE2L2* as topologically important nodes within dense subnetwork structures. Betweenness centrality identified *ESR1*, *TNF*, and *TP53* as major information-flow mediators, whereas closeness centrality confirmed *IL6* and *TNF* as highly connected targets within the network. Taken together, these results suggest that the predicted DAP target network is organized around inflammatory, hormonal, and stress responses and cell survival-related hubs.

### 2.4. Functional Enrichment Reveals Four Skin Aging-Related Mechanistic Axes

**Figure 4:**
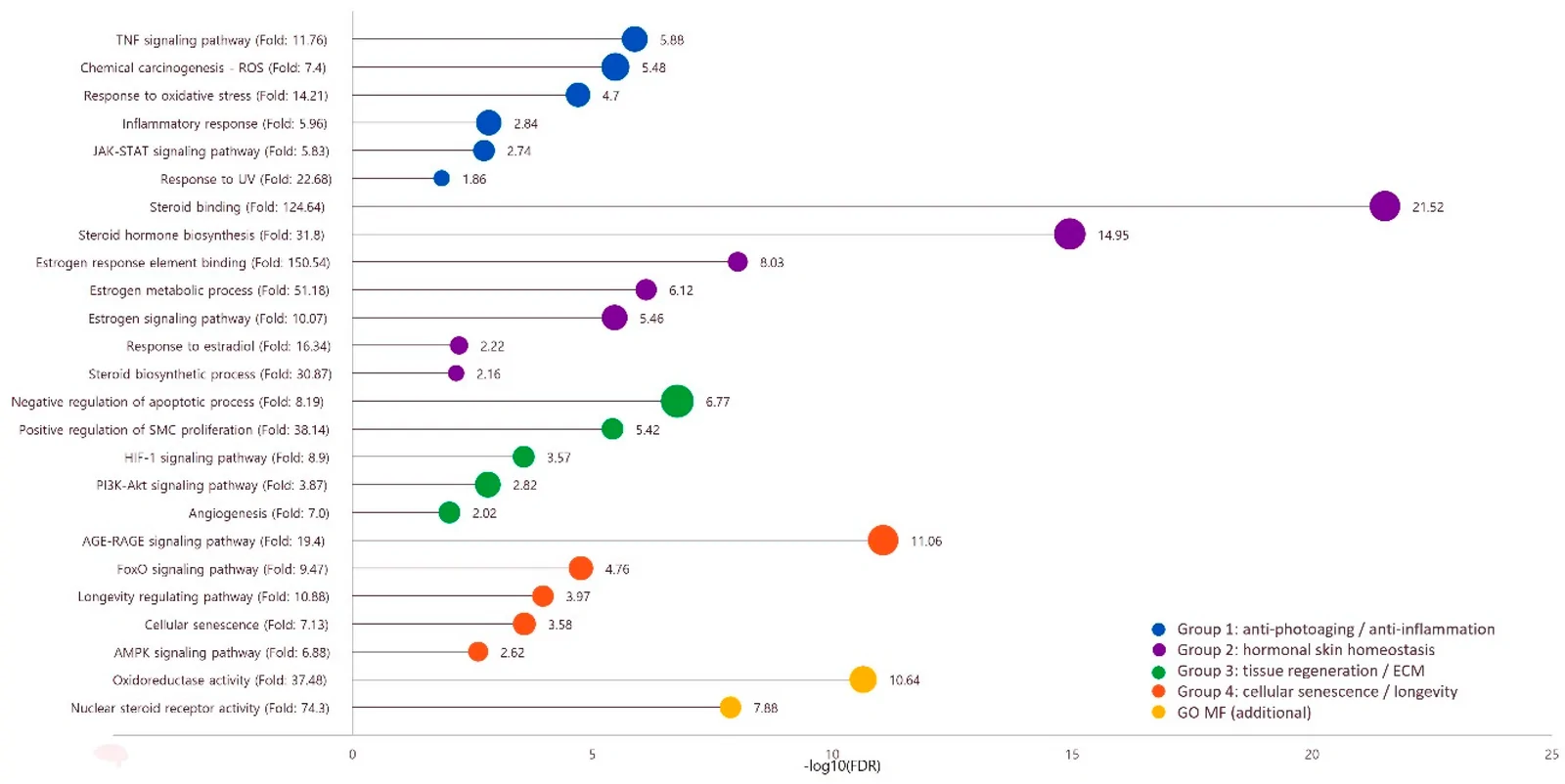
Gene Ontology and Kyoto Encyclopedia of Genes and Genomes enrichment analysis of predicted DAP targets. Representative enriched terms (n = 17) selected from DAVID analysis (FDR < 0.05) are grouped into four predicted mechanistic axes and an additional GO MF category. The x-axis represents enrichment significance as −log10(FDR); bubble size indicates gene count; numerical labels indicate fold enrichment values. Full enrichment statistics are provided in Table S4. ECM, extracellular matrix; GO MF, Gene Ontology molecular function; FDR, false discovery rate.

Gene Ontology (GO) and Kyoto Encyclopedia of Genes and Genomes (KEGG) enrichment analyses were performed using DAVID, with 70 final target genes as inputs. A total of 341 terms were significantly enriched at a false discovery rate (FDR) < 0.05. Seventeen representative terms were selected based on FDR significance, fold enrichment, gene count, and biological relevance to skin aging-related mechanisms (Table 2 and Figure 4). The full enrichment results are presented in Table S4.

#### 2.4.1. Inflammatory and Oxidative Stress-Related Axis

Significantly enriched terms included the TNF signaling pathway, JAK-STAT signaling pathway, chemical carcinogenesis-ROS, inflammatory response, response to oxidative stress, and response to UV radiation. These terms were associated with targets such as *TNF*, *IL6*, *PTGS2*, *NFE2L2*, *HMOX1*, *MAPK8*, and *STAT1*, suggesting that the predicted DAP target network was closely related to inflammatory and oxidative stress-related processes implicated in photoaging and inflammation.

#### 2.4.2. Hormonal Skin Homeostasis-Related Axis

Estrogen- and steroid-related terms were prominently enriched, including steroid hormone biosynthesis, estrogen signaling pathway, estrogen metabolic process, estradiol response, steroid binding, estrogen response element binding, and nuclear steroid receptor activity. These enrichments were associated with hormone-related targets, such as *ESR1*, *ESR2*, *AR*, *PGR*, *CYP19A1*, *CYP11B2*, and *SHBG*. Because these results were derived from network and enrichment analyses, they should be interpreted as predicted hormone-related mechanistic signals rather than as evidence of systemic endocrine activity.

#### 2.4.3. Tissue Repair and ECM Remodeling-Related Axis

Terms related to tissue repair and remodeling included PI3K–Akt signaling pathway, HIF-1 signaling pathway, negative regulation of apoptotic processes, angiogenesis, and proliferation-related biological processes. Targets such as *AKT1*, *EGFR*, *TGFB1*, *COL1A1*, *MMP13*, and *PIK3CA* contribute to this axis. These findings suggest that DAP-related compounds may be associated with pathways involved in cell survival, tissue repair, and ECM regulation.

#### 2.4.4. Cellular Senescence and Longevity-Related Axis

Cellular senescence, the *FoxO* signaling pathway, the longevity-regulating pathway, the *AMPK* signaling pathway, and the *AGE*–*RAGE* signaling pathway were significantly enriched. The representative targets included *TP53*, *SIRT1*, *FOXO3*, *AKT1*, *CASP3*, and *PPARA*. These results suggested that the predicted DAP network may intersect with cellular stress adaptation and senescence-related regulatory pathways.

### 2.5. ADME and Preliminary Route-Relevant Safety Profiling of the Injectable Formulation

#### 2.5.1. Supplementary ADME Profiling of Candidate Compounds

ADME profiling was performed using SwissADME for all 165 candidate compounds, including 144 compounds from the eight-component base formulation and 21 compounds from the three additional skin-oriented components. In the base formulation, 118 compounds showed a high predicted gastrointestinal absorption, whereas 26 showed a low predicted gastrointestinal absorption. In contrast, among the three additional skin-oriented components, only 6 of the 21 compounds showed high predicted gastrointestinal absorption, whereas 15 compounds showed low predicted gastrointestinal absorption. Across the entire DAP compound set, 41 compounds exhibited low predicted gastrointestinal absorption and 26 had topological polar surface area values greater than 140 Å^2^.

The high proportion of low predicted gastrointestinal absorption of the three additional components was largely attributable to their physicochemical properties. Ginsenoside Rb1 showed the highest topological polar surface area among all the DAP-related compounds and had two Lipinski rule violations. Ginsenosides Rg3, Re, and Rd also exhibited high topological polar surface areas and multiple Lipinski violations. Asiaticoside and madecassoside from *C. asiatica* similarly show high topological polar surface areas and three Lipinski violations. In contrast, several steroid hormones from the Hominis Placenta showed high predicted gastrointestinal absorption and low topological polar surface area values, but these compounds may undergo extensive first-pass metabolism after oral administration. These findings provide a supplementary physicochemical context for the non-oral injectable nature of DAP. However, predicted oral absorption does not directly reflect dermal or subcutaneous pharmacokinetics, and local retention, systemic exposure, and tissue distribution require further experimental investigation.

#### 2.5.2. Single-Dose Subcutaneous Toxicity Evaluation Provides Preliminary Route-Relevant Safety Information

Because DAP is designed as an injectable formulation, preliminary route-relevant safety was evaluated using a single-dose subcutaneous injection toxicity study in Sprague-Dawley rats. The study was conducted in compliance with GLP regulations and relevant toxicological test guidelines. Male and female rats were assigned to DAP-treated and saline vehicle control groups. DAP was administered via subcutaneous injection at a dose of 1.0 mL/head. Mortality, clinical signs, body weight, gross necropsy findings, and histopathological findings at the injection site were assessed over a 14-day observation period.

No mortality occurred in any of the animals during the observation period. No abnormal clinical signs were observed in the DAP-treated group. Transient decreases in body weight were observed on day 2 in both the control and DAP-treated groups, followed by recovery by day 4; no statistically significant differences in body weight were detected between the groups throughout the observation period. Gross necropsy revealed no DAP findings. The overall single-dose toxicity findings are summarized in Table 3.

**Figure 5:**
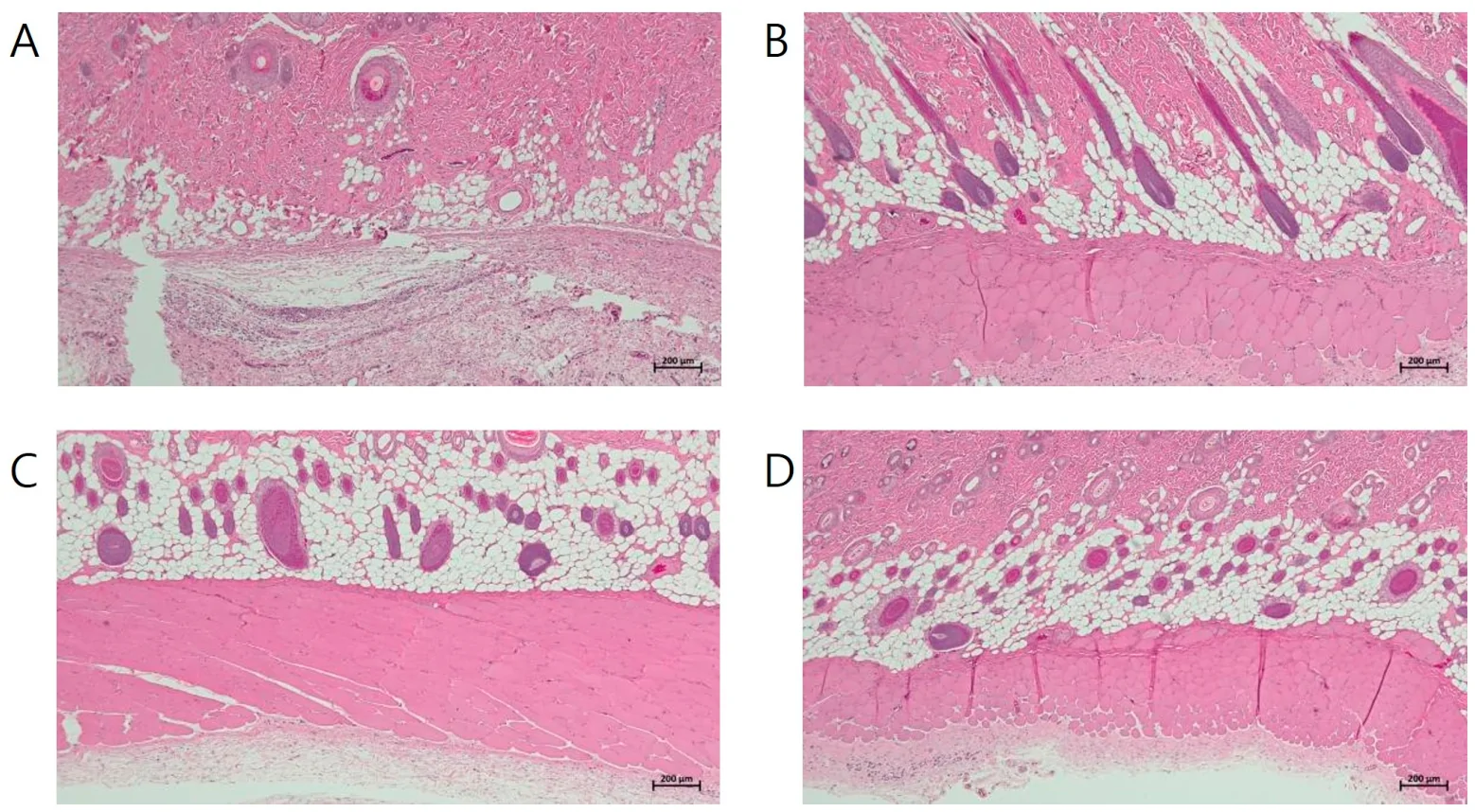
Representative histopathological findings at the subcutaneous injection site following single-dose subcutaneous administration of DAP in Sprague–Dawley rats. The injection site was the nape of the neck. (**A**) Male vehicle control group (group 1), showing minimal mononuclear cell infiltration in the subcutis. (**B**) Male DAP-treated group (group 2), showing normal tissue structure. (**C**) Female vehicle control group (group 1), showing normal tissue structure. (**D**) Female DAP-treated group (group 2), showing normal tissue structure. The isolated finding in one male vehicle control animal was interpreted as a nonspecific injection procedure-related response rather than a DAP-related histopathological change. Hematoxylin and eosin stain, scale bar = 200 μm. DAP, dong-an pharmacopuncture.

Histopathological examination of the subcutaneous injection site revealed minimal mononuclear cell infiltration in the subcutis of one male in group 1 (saline vehicle control). This finding was considered incidental and related to the injection procedure rather than to DAP administration, because it was observed only in the vehicle control group and was absent in the DAP-treated group. No histopathological abnormalities were observed at the injection sites in any of the remaining animals (Figure 5).

Based on these findings, the approximate lethal dose of DAP following single-dose subcutaneous injection was determined to be greater than 1.0 mL/head under the conditions of this study. These results provide preliminary evidence suggesting no overt acute toxicity or DAP-related injection site histopathological abnormalities under the tested conditions. However, this single-dose study did not establish repeated-dose safety, sensitization potential, endocrine safety, or clinical safety in humans.

## 3. Discussion

### 3.1. Principal Findings and Systems-Level Interpretation

This study evaluated the mechanistic and preliminary safety profiles of DAP, a multicomponent natural product-based injectable formulation that targets skin aging-related pathways. By integrating compound screening, target prediction, disease-target intersection, H-C-T network construction, PPI topology, enrichment analysis, ADME profiling, and a GLP-compliant single-dose toxicity study, we identified a system-level framework for the predicted biological actions of DAP. The final network comprised 70 skin aging-related targets and 225 compound–target interactions, with ten core hub targets (*TNF*, *IL6*, *ESR1*, *TP53*, *AKT1*, *PPARG*, *EGFR*, *PTGS2*, *CASP3*, and *PPARA*) identified across topological analyses.

These hub targets suggest that the predicted DAP network is not centered on a single molecular mechanism but instead spans several biological domains relevant to skin aging. Inflammatory mediators such as *TNF* and *IL6* [10,24,25], hormone-related targets such as *ESR1* and stress response, survival-related targets such as *AKT1* and *TP53* [22,26], and lipid/metabolic regulators such as *PPARG* and *PPARA* are all represented within the core network. In addition, *SIRT1* and *NFE2L2* [7] have been highlighted as topologically important nodes in dense subnetwork structures. These findings support the interpretation of DAP as a multicomponent formulation, whose predicted target profile may be better understood through a multiaxis framework rather than a single-target model. However, these results remain computational and hypothesis-generating and should not be interpreted as evidence of clinical efficacy.

### 3.2. Four Predicted Mechanistic Axes Related to Skin Aging

Functional enrichment analysis delineated four major mechanistic axes potentially relevant to skin aging: inflammatory/oxidative stress regulation, hormonal skin homeostasis, tissue repair/ECM remodeling, and cellular senescence-related regulation. The inflammatory and oxidative stress-related axis was supported by the enrichment of TNF signaling, JAK-STAT signaling, inflammatory response, response to oxidative stress, and response to UV. These findings are consistent with the recognized roles of UV-induced ROS, inflammatory cytokines, and oxidative stress responses in photoaging and inflammaging [1,10]. Targets such as *TNF*, *IL6*, *PTGS2*, *NFE2L2*, *HMOX1*, *MAPK8*, and *STAT1* contribute to this axis, suggesting that the predicted DAP network intersects with inflammatory and antioxidant response pathways relevant to skin aging [7,24,25].

The hormonal skin homeostasis-related axis was characterized by the enrichment of estrogen- and steroid-related terms, including estrogen signaling pathway, estrogen metabolic process, steroid binding, estrogen response element binding, and nuclear steroid receptor activity. These enrichments were associated with targets, such as *ESR1*, *ESR2*, *AR*, *PGR*, *CYP19A1*, *CYP11B2*, and *SHBG*. Because estrogen signaling is involved in collagen homeostasis, epidermal function, and skin barrier regulation, this enrichment may be biologically relevant to age-related changes in the skin, particularly in the context of hormonal decline [3,4]. However, these estrogen-related findings must be interpreted strictly as network-level mechanistic signals; they do not demonstrate systemic endocrine activity, local estrogenic effects in skin tissue, or hormonal safety of DAP after repeated administration.

The tissue repair and ECM remodeling-related axis includes PI3K-Akt signaling, HIF-1 signaling, negative regulation of apoptosis, angiogenesis, and proliferation processes. Targets such as *AKT1*, *EGFR*, *TGFB1*, *COL1A1*, *MMP13*, and *PIK3CA* contribute to this axis. These findings suggest that DAP-related compounds may be associated with pathways involved in cell survival, tissue repair, collagen regulation, and matrix remodeling. In particular, *C. asiatica*-derived triterpenoids, such as asiaticoside and madecassoside, have been linked in previous studies to collagen synthesis, wound healing, and MMP regulation, which may provide a plausible basis for the ECM-related signals observed in the network [15,16].

**Figure 6:**
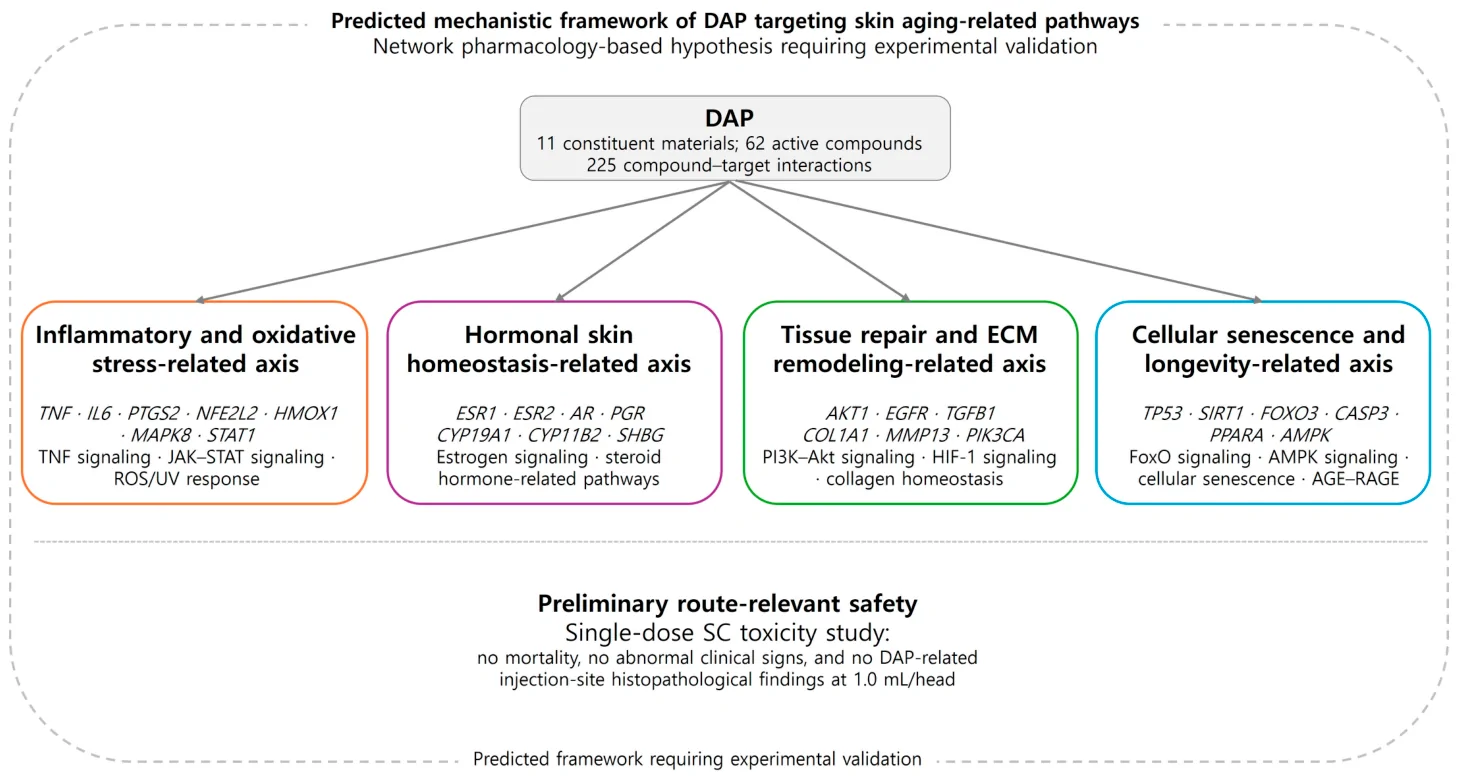
Predicted mechanistic framework of DAP targeting skin aging-related pathways. Network pharmacology analysis of DAP identified 62 active compounds, 70 final targets, and 225 compound–target interactions. Functional enrichment analysis suggested four mechanistic axes related to skin aging: inflammatory and oxidative stress-related regulation, hormonal skin homeostasis, tissue repair, ECM remodeling, and cellular senescence/longevity-related regulation. The representative targets and pathways are shown for each axis. A Good Laboratory Practice-compliant single-dose subcutaneous toxicity study revealed no mortality, abnormal clinical signs, or DAP-related injection site histopathological findings at 1.0 mL/head under the tested conditions, thus providing preliminary route-relevant safety information. All mechanistic findings are computational predictions and require experimental validation in skin-relevant models; however, this study did not establish the clinical anti-aging efficacy of DAP. DAP, dong-an pharmacopuncture; ECM, extracellular matrix; SC, subcutaneous.

The cellular senescence and longevity-related axis were supported by the enrichment of cellular senescence, *FoxO* signaling, the longevity-regulating pathway, *AMPK* signaling, and *AGE*–*RAGE* signaling. The representative targets included *TP53*, *SIRT1*, *FOXO3*, *AKT1*, *CASP3*, and *PPARA*. These pathways are involved in cellular stress adaptation, the DNA damage response, metabolic regulation, and senescence-associated inflammatory signaling [5]. The involvement of *SIRT1*, *AMPK*, and *FoxO*-related pathways suggests a possible connection between DAP-related compounds and the stress-resilience pathways implicated in skin aging. Nevertheless, direct validation in skin-relevant cells or animal models is required before any conclusions can be drawn regarding senescence modulation. The enrichment of *SIRT1*, *AMPK*, and *FoxO*-related pathways may also be viewed in relation to the broader NAD (nicotinamide adenine dinucleotide)-dependent longevity network currently discussed in skin aging research, in which NAD homeostasis has been linked to SIRT1-mediated cellular stress adaptation and epidermal function [26,27]. However, DAP was not evaluated as an NAD precursor or booster in this study, and the relevance of this mechanistic axis requires direct experimental validation. Taken together, these findings provide a network-level basis for a predicted multiaxis mechanistic framework of DAP in skin aging-related pathways; however, further experimental validation in skin-relevant models is required (Figure 6).

Previous network pharmacology studies of natural product-based anti-aging interventions have similarly identified multitarget networks involving inflammatory, oxidative stress, ECM remodeling, and cell survival-related pathways. For example, a network pharmacology study of Hibiscus mutabilis leaf extract in UV-induced photoaging identified *AKT1*, *TNF*, *STAT3*, *MMP9*, and *EGFR* as core targets and subsequently supported selected predictions in cellular and animal models [28]. The present study shares this systems-level perspective but is distinguished by its focus on an injectable eleven-component natural product formulation and by the additional prominence of hormone-related and senescence/longevity-related network signals in the predicted target profile. These comparisons do not imply comparative or superior efficacy of DAP relative to other formulations; rather, they provide contextual support for the use of network approaches to prioritize mechanistic hypotheses in multicomponent anti-aging formulations.

### 3.3. Contribution of the Three Skin-Oriented Additional Components

The component-group-level summary provides a descriptive view of how different constituent groups contribute to the predicted DAP network. The three additional skin-oriented components, *C. asiatica*, Hominis Placenta, and *P. quinquefolius*, were associated with targets related to hormone-related signaling, ECM/collagen remodeling, senescence-related regulation, and melanogenesis-related regulation, whereas inflammatory and oxidative stress-related targets were distributed across the broader eleven-component formulation. This suggests that DAP may cover several biologically relevant axes of skin aging at the network level.

These findings provide a mechanistic rationale for evaluating DAP as an integrated eleven-component formulation. *C. asiatica* may contribute primarily to ECM/collagen remodeling through compounds such as asiaticoside, madecassoside, quercetin, kaempferol, and ursolic acid [15,16]. The Hominis Placenta may contribute to hormone-related and regenerative signaling through steroid hormones, melatonin, and other bioactive molecules [17,18]. *P. quinquefolius* may contribute to antioxidant activity, cell survival, metabolic stress, and melanocyte-related pathways through ginsenosides and their related constituents [19,20]. From a Korean medicine perspective, the inclusion of Hominis Placenta, *P. quinquefolius*, and *C. asiatica* is also consistent with traditional concepts related to tonification, regeneration, and tissue repair. However, these traditional concepts were used only as contextual backgrounds, and the present study focused on predicted molecular networks. A previous clinical study of cultivated wild ginseng pharmacopuncture reported improvements in wrinkle-related outcomes, providing a limited clinical context for ginseng-derived pharmacopuncture approaches; however, these findings cannot be directly extrapolated to DAP [14]. Thus, the three skin-oriented components appeared to map onto complementary skin aging-related domains. Importantly, this component-level analysis was not intended to infer additive, synergistic, superior, or comparative efficacies. Instead, it provides a hypothesis-generating map of how the predicted targets are distributed across the DAP constituent groups.

Although the eight-component base formulation has previously been analyzed in a different disease context [21], prior analyses did not establish its dermatological efficacy and should not be used as direct evidence for DAP. Therefore, the present study evaluated DAP as a distinct modified formulation for skin aging. The observed shift in the predicted target distribution toward hormone-related signaling, ECM remodeling, and senescence-related regulation remains computational and requires experimental confirmation.

### 3.4. Supplementary ADME Profiling and Physicochemical Context

These ADME findings provide a supplementary physicochemical context for interpreting DAP as a non-oral injectable formulation. Among the three skin-oriented components, 71.4% showed a low predicted gastrointestinal absorption. This profile was largely driven by the physicochemical properties of major constituent classes, including ginsenosides and *Centella*-derived triterpenoids, which show high topological polar surface area values and multiple Lipinski rule violations, parameters associated with limited oral drug-likeness (DL) and membrane permeability [29,30,31]. These results suggested that oral delivery may not fully reflect the expected exposure profiles of several DAP-related constituents.

Therefore, the injectable route may be relevant for future studies investigating local exposure to compounds derived from poorly absorbed natural products. However, this interpretation has several important limitations. SwissADME is primarily designed to evaluate physicochemical properties, oral pharmacokinetic parameters, DL, and medicinal chemistry friendliness and does not directly estimate subcutaneous absorption, dermal retention, local tissue distribution, or systemic exposure after injection [29]. In addition, the concentrations and pharmacokinetic behaviors of most of the predicted active constituents in the final injectable formulation remain to be determined. As a supplementary marker-based confirmation, HPLC analysis confirmed asiaticoside in the DAP batch used for toxicity testing. However, this single-marker result did not establish a quantitative profile or biological activity of the full multicomponent formulation. Future studies should expand the quantitative profiling of additional marker compounds and investigate their local tissue distribution and pharmacokinetic behavior after subcutaneous administration.

### 3.5. Preliminary Route-Relevant Safety Findings

Because DAP is administered as an injectable formulation, route-relevant safety evaluation is important in addition to mechanistic prediction. Single-dose toxicity studies are commonly used as an initial nonclinical approach to evaluate acute toxicity and estimate the approximate lethal dose of pharmacopuncture preparations in rodent models [32,33]. In a GLP-compliant single-dose subcutaneous toxicity study, no mortality, abnormal clinical signs, abnormal gross necropsy findings, or injection site histopathological findings attributable to DAP were observed at 1.0 mL/head in Sprague-Dawley rats. The approximate lethal dose was determined to be greater than 1.0 mL/head under the conditions of the study. These findings provide preliminary evidence suggesting no overt acute toxicity or injection site histopathological abnormalities under the tested conditions.

Nevertheless, the safety findings should be interpreted cautiously. Single-dose toxicity studies cannot establish repeated-dose safety, cumulative local tissue responses, sensitization potential, reproductive or endocrine safety, genotoxicity, or clinical safety in humans [34]. Given the enrichment of estrogen- and steroid-related pathways identified in the network analysis, future repeated-dose safety studies should include endocrine-related endpoints and systemic exposure assessments after subcutaneous administration, rather than relying solely on acute local tolerability data. Local tolerability should also be assessed under administrative conditions closely aligned with potential clinical administration.

### 3.6. DAP in the Context of Contemporary Injectable Skin Rejuvenation Research

Contemporary injectable skin rejuvenation approaches include diverse formulation categories, such as hyaluronic acid-based skin boosters, polynucleotide- or nucleotide-based preparations, polycomponent mesotherapy formulations, placental extract injections, platelet-rich plasma or extracellular vesicle-based preparations, and collagen biostimulators [35,36,37]. These approaches differ substantially in composition, intended mechanism, tissue persistence, and risk profile, and are generally discussed in relation to hydration, ECM support, tissue repair, regenerative signaling, inflammatory modulation, or controlled collagen biostimulation. The present study did not directly compare DAP with these injectable approaches, nor did it provide evidence of comparative efficacy or safety. However, placing DAP within this broader formulation landscape helps to clarify its position as a soluble, multicomponent, natural product-based injectable preparation.

In relation to these formulation categories, several predicted DAP targets overlapped with biological processes commonly discussed in injectable skin rejuvenation research. For example, ECM-related targets such as *COL1A1* and *TGFB1*, inflammatory mediators such as *TNF* and *IL6*, and antioxidant response-related targets such as *NFE2L2* are relevant to collagen regulation, tissue repair, inflammatory modulation, and oxidative stress control, which are also discussed in relation to nucleotide-based preparations, polycomponent mesotherapy formulations, placental extract-based approaches, and other regenerative injectables [35,36,37]. At the same time, the predicted DAP network additionally highlighted hormone-related signaling and cellular senescence/longevity-related mechanisms, including *ESR1*, *SIRT1*, and *FoxO*-, *AMPK*-, and *AGE*–*RAGE*-related pathways [26,27,38]. These findings do not indicate that DAP is superior to existing injectable preparations. Rather, they suggest that DAP may represent a distinct soluble, multicomponent, natural product-based injectable formulation with a broader predicted network distribution that warrants experimental validation.

### 3.7. Limitations and Future Directions

This study has several limitations. First, compound–target interactions predicted by STITCH and supported by literature curation may not reflect the actual binding, activity, or pharmacological relevance at physiologically achievable concentrations. Second, the GeneCards-derived disease target set may include heterogeneous and non-specific targets related to broad skin aging terms, which can influence the enrichment results. Third, manual supplementation and filtering of targets introduced investigator judgment, but this process was applied to retain biologically relevant skin age-related targets that were not captured by database prediction. Fourth, ADME profiling is based on the predicted oral absorption parameters and does not provide direct information regarding subcutaneous pharmacokinetics, dermal retention, tissue distribution, or systemic exposure. Fifth, the toxicity evaluation was limited to a single-dose subcutaneous study. Repeated-dose toxicity, local irritation, sensitization, genotoxicity, reproductive toxicity, endocrine-related safety, and systemic toxicity should be evaluated in future studies. Sixth, molecular docking or other target-binding validation approaches were not performed. Future studies incorporating validated docking, biophysical assays, or cell-based target validation could strengthen the compound–target evidence. Seventh, the predicted pathways require validation in skin-relevant experimental models, including keratinocytes, dermal fibroblasts, melanocytes, three-dimensional skin equivalents, UV-induced photoaging models, and in vivo skin aging models. In addition, the clinical efficacy and safety cannot be inferred from the present study and should be evaluated through structured clinical trials using standardized skin aging outcomes and safety monitoring. Eighth, this study did not include an active comparator or dose–response evaluation for efficacy. Although a saline vehicle control was included in the single-dose toxicity assessment, no active comparator was evaluated for skin aging-related efficacy. Accordingly, comparative efficacy, optimal dosing, and dose–response relationships cannot be inferred from the present data. Ninth, chemical standardization of the multicomponent formulation remains preliminary. Although asiaticoside was confirmed by HPLC-UV as a marker compound in the DAP batch used for toxicity testing, single-marker confirmation is insufficient to establish quantitative composition, batch-to-batch consistency, or the biological contribution of the full formulation. Future studies should incorporate multi-marker quantitative profiling and batch consistency assessment. Tenth, the inherent limitations of the databases used in this study should be acknowledged. STITCH predictions depend on the availability and quality of existing experimental, curated, and text-mining evidence and may therefore preferentially represent well-studied compounds and targets. GeneCards-derived disease targets are aggregated from multiple sources and depend on the search terms applied, potentially introducing non-specific targets. DAVID enrichment results are influenced by input gene-set composition and annotation coverage, and enriched pathways may reflect annotation density rather than biological significance. The network and enrichment results should therefore be interpreted as database-dependent, hypothesis-generating predictions rather than direct evidence of biological activity. Finally, the toxicity evaluation did not address immunogenicity, which is particularly relevant for a multicomponent injectable formulation intended for repeated clinical use. Future safety studies should include immunogenicity assessment in addition to repeated-dose toxicity, cumulative local tolerance, and systemic safety endpoints.

Overall, this study provides a system-level mechanistic framework and preliminary route-relevant safety information for DAP as a multicomponent natural product-based injectable formulation that targets skin aging-related pathways. These findings support further experimental validation of the predicted inflammatory, oxidative stress-related, hormonal, ECM remodeling, and senescence-related mechanisms, as well as more comprehensive safety and pharmacokinetic evaluations before further translational development.

## 4. Methods

### 4.1. Active Compound Collection and ADME Profiling

The active compounds in the 11 constituent materials of DAP were identified through integrated database mining and systematic literature-based curation. For plant-derived constituent materials with database coverage, compounds were retrieved from the TCMSP (https://www.tcmsp-e.com/tcmsp.php, accessed on 1 May 2026) [39] and the HERB database (http://herb.ac.cn/, accessed on 1 May 2026) using DL (≥0.18) as a primary screening criterion. For animal- or biologically derived constituent materials, including *Moschus*, *Fel ursi*, *Calculus bovis*, and Hominis Placenta, bioactive compounds were identified through a systematic literature curation based on documented pharmacological activity. The compounds from *P. quinquefolius* and *C. asiatica* were curated based on published phytochemical analyses and pharmacological studies in skin-related contexts. The final set of active compounds was used for network construction, whereas the broader candidate compound set identified prior to target-based filtering was retained for ADME profiling.

Physicochemical and ADME profiling were performed using SwissADME (https://www.swissadme.ch/, accessed on 1 May 2026) [29] for all candidate compounds identified prior to target-based filtering. The parameters evaluated included molecular weight, topological polar surface area, gastrointestinal absorption prediction, Lipinski’s rule-of-five compliance, blood–brain barrier permeability, and bioavailability score. Compounds exhibiting low predicted gastrointestinal absorption or high topological polar surface area values were specifically examined in the context of pharmacopuncture delivery, as direct injection bypasses gastrointestinal absorption barriers and hepatic first-pass metabolism, potentially allowing the investigation of local tissue exposure of selected constituents at dermal target sites.

### 4.2. Target Prediction and Disease Target Collection

Compound–target interactions were predicted using the STITCH database (v5.0, https://stitch-db.org/, combined confidence score ≥ 0.4) [40] with compounds submitted in two groups corresponding to the eight-component base formulation and the three additional skin-oriented components. All predicted targets were mapped to official gene symbols for standardization. Skin aging-related disease targets were collected from GeneCards (https://www.genecards.org/, accessed on 1 May 2026) using the following seven search terms: “skin anti-aging,” “skin elasticity,” “facial aging,” “cellular senescence,” “cellular aging,” “tissue regeneration,” and “wound healing.” The intersection between compound-predicted and disease targets was identified using Venn diagram analysis. Eight additional targets with strong literature-based compound–target evidence not captured by STITCH were manually supplemented. Quality filtering excluded targets without compound-level interaction evidence, housekeeping genes, noise targets, or PPI-isolated nodes. Three biologically relevant targets were added based on STITCH or literature: *HMOX1*, *PTGS2*, and *MMP13*. The final set of 70 targets was consistently used in all downstream analyses.

### 4.3. Network Construction and Visualization

The H-C-T network was constructed and visualized using Cytoscape (v3.10.1, https://cytoscape.org/) [41]. The PPI network was constructed using STRING (v12.0, https://string-db.org/, *Homo sapiens*, confidence ≥ 0.4) [42]. Network topology was analyzed using the NetworkAnalyzer plugin for degree, betweenness, and closeness centralities. Hub targets were identified using the CytoHubba plugin with the degree centrality algorithm. The top 10 targets by degree score were defined as core hub targets.

### 4.4. GO and KEGG Enrichment Analysis

GO and KEGG enrichment analyses were performed using DAVID (v2023, https://david.ncifcrf.gov/, *H. sapiens* background) [43] with 70 target genes as input. Terms with FDR < 0.05 (Benjamini-Hochberg correction) were considered statistically significant. Seventeen representative terms were selected for mechanistic interpretation based on statistical significance, fold enrichment, gene count, and direct biological relevance to skin aging-related targets and organized into four pharmacological axes. The full enrichment results are provided in Supplementary Materials.

### 4.5. Test Substance Preparation and Single-Dose Subcutaneous Toxicity Study

The DAP formulations used in this study (lot no. NN0726001) were manufactured and provided by the Namsangcheon external herbal dispensary facility (Yongin, Republic of Korea). The test substance was supplied as a clear, light brown liquid in 2 mL transparent vials and stored at room temperature (1–30 °C), protected from light, until use. The formulation was supplied by the manufacturer and used without further preparation. For supplementary marker-based quality confirmation, the DAP batch used in this study was analyzed for asiaticoside, a major marker compound of *C. asiatica*, using HPLC-UV. Analyses for asiaticoside quantification was performed using an Agilent 1260 HPLC system (Seoul, Republic of Korea) with UV detection at 206 nm under in-house analytical conditions. The asiaticoside content was calculated using a certified asiaticoside standard and was expressed in milligrams per vial.

The single-dose subcutaneous injection toxicity study of DAP was conducted at HLB BioCode Co., Ltd. (Suwon, Republic of Korea; study no. C26RA-075G), in compliance with GLP regulations [44] and Ministry of Food and Drug Safety Toxicological Test Guidelines [34]. Specific pathogen-free Sprague–Dawley rats (7 weeks old at receipt; ORIENTBIO Co., Ltd., Seongnam, Republic of Korea) were used. Following a 3-day quarantine and 7-day acclimation period, healthy animals (8 weeks of age; n = 5/sex/group) were assigned to the DAP-treated group (1.0 mL/head) and saline vehicle control group (0.9% saline, 1.0 mL/head) by zigzag body weight stratification. DAP or saline was administered as a single subcutaneous injection into the nape of the neck using a 26-gauge disposable syringe following disinfection with 70% ethanol. A dose of 1.0 mL/head was selected as the high-dose level based on the proposed clinical application volume.

All animals were observed for mortality and clinical signs at 30 min and 1, 2, 3, and 4 h post-dosing on day 1 and once daily for 14 days thereafter. Body weights were recorded on days 1, 2, 4, 8, and 15, using a calibrated electronic balance (BCE2202-1SKR; Sartorius, Göttingen, Germany). On day 15, all surviving animals were euthanized by CO_2_ inhalation, followed by exsanguination from the abdominal aorta, and complete gross necropsy was performed. The injection site and surrounding subcutaneous tissues were excised, fixed in 10% neutral-buffered formalin (Merck, Darmstadt, Germany), processed onto hematoxylin and eosin-stained (Merck, Darmstadt, Germany) tissue slides, and examined histopathologically for cell infiltration, necrosis, and edema.

Statistical analysis of body weight data was performed using Student’s *t*-test or Welch’s *t*-test following Levene’s test for homogeneity of variance, with *p* < 0.05 considered statistically significant (IBM SPSS Statistics, version 24; IBM Corp., Armonk, NY, USA).

## 5. Conclusions

This study provides the systems-level mechanistic and preliminary safety profile of DAP, a multicomponent natural product-based injectable formulation that targets skin aging-related pathways. Network pharmacology analysis identified 62 active compounds, 70 final targets, and 225 compound–target interactions, and functional enrichment suggested four predicted mechanistic axes: inflammatory and oxidative stress regulation, hormonal skin homeostasis-related signaling, tissue repair and ECM remodeling, and cellular senescence-related regulation. ADME profiling supported the rationale for investigating DAP as a non-oral injectable formulation, particularly the poorly absorbed constituents of the three skin-oriented components. A GLP-compliant single-dose subcutaneous toxicity study showed no mortality, abnormal clinical signs, or histopathological findings attributable to DAP 1.0 mL/head, providing preliminary route-relevant safety information under the tested conditions. These findings support further experimental validation of the predicted mechanisms, together with pharmacokinetics, repeated-dose safety, and clinical studies before translational application. Specifically, future experimental validation should include skin-relevant cellular models, such as UV-stressed keratinocyte and dermal fibroblast assays evaluating collagen synthesis, MMP activity, and senescence-related endpoints, followed by efficacy studies in appropriate in vivo photoaging or skin-aging animal models, together with pharmacokinetic characterization of key constituents after subcutaneous administration.

## Figures and Tables

**Table 1:** Composition of DAP: constituent materials, sources, formulation groups, and representative active compounds.

No.	Constituent Material	Botanical/Biological Source	Formulation Group	Representative Active Compounds
1	*Moschus*	*Moschus moschiferus* (musk deer)	Eight-component base	Muscone
2	*Fel ursi*	*Ursus arctos* (bear bile)	Eight-component base	UDCA, TUDCA
3	*Calculus bovis*	*Bos taurus* (bovine gallstone)	Eight-component base	Bilirubin, cholic acid, taurocholic acid
4	*Scutellariae radix*	*Scutellaria baicalensis* Georgi	Eight-component base	Baicalin, baicalein, wogonin, quercetin
5	*Phellodendri cortex*	*Phellodendron amurense* Rupr.	Eight-component base	Berberine, palmatine
6	*Pulsatillae radix*	*Pulsatilla koreana* (Yabe ex Nakai) Nakai ex Mori	Eight-component base	Oleanolic acid, anemonin
7	*Sophorae tonkinensis radix*	*Sophora tonkinensis* Gagnep.	Eight-component base	Oxymatrine, matrine, genistein
8	*Aucklandiae radix*	*Aucklandia lappa* Decne.	Eight-component base	Costunolide, dehydrocostus lactone
9	*Panax quinquefolius*	*Panax quinquefolius* L.	Skin-oriented addition	Ginsenosides Rb1, Rg1, Rg3, Re, Rd
10	Hominis Placenta	Human placenta	Skin-oriented addition	Steroid hormones, melatonin
11	*Centella asiatica*	*Centella asiatica* (L.) Urb.	Skin-oriented addition	Asiaticoside, madecassoside, quercetin, kaempferol, ursolic acid

UDCA, ursodeoxycholic acid; TUDCA, tauroursodeoxycholic acid. Botanical and biological source names follow current pharmacopoeial conventions. Representative compounds are selected from those identified in Table S1.

**Table 2:** Summary of four predicted mechanistic axes related to skin aging.

Predicted Mechanistic Axis	Representative Enriched Terms	Representative Targets	Network-Level Interpretation
Inflammatory and oxidative stress-related regulation	*TNF* signaling pathway; response to oxidative stress; chemical carcinogenesis–ROS; response to UV	*TNF*, *IL6*, *PTGS2*, *NFE2L2*, *HMOX1*, *MAPK8*, *STAT1*	Potential involvement in inflammatory and oxidative stress-related processes implicated in photoaging and inflammaging
Hormonal skin homeostasis	Steroid hormone biosynthesis; estrogen signaling pathway; steroid binding; estrogen response element binding	*ESR1*, *ESR2*, *AR*, *PGR*, *CYP19A1*, *CYP11B2*, *SHBG*	Predicted association with hormone-related signaling relevant to collagen homeostasis, epidermal function, and skin barrier regulation
Tissue repair and ECM remodeling	*PI3K*–*Akt* signaling pathway; *HIF-1* signaling pathway; negative regulation of apoptotic process; positive regulation of SMC proliferation	*AKT1*, *EGFR*, *TGFB1*, *COL1A1*, *MMP13*, *PIK3CA*	Potential association with cell survival, tissue repair, angiogenesis, and ECM regulation
Cellular senescence and longevity-related regulation	Cellular senescence; *FoxO* signaling pathway; *AGE*–*RAGE* signaling pathway; *AMPK* signaling pathway	*TP53*, *SIRT1*, *FOXO3*, *CASP3*, *PPARA*, *AKT1*	Predicted involvement in cellular stress adaptation, senescence-related regulation, and metabolic/longevity-associated pathways
Additional molecular function terms	Oxidoreductase activity; nuclear steroid receptor activity	*NFE2L2*, *HMOX1*, *ESR1*, *ESR2*, *AR*, *PGR*	Additional functional support for oxidative stress-related and steroid receptor-related signaling

Representative terms were selected from Gene Ontology and Kyoto Encyclopedia of Genes and Genomes enrichment analyses at FDR < 0.05 based on statistical significance, gene count, fold enrichment, and biological relevance to skin aging-related mechanisms. Full enrichment statistics, including count, FDR, and fold enrichment, are provided in Table S4. ROS, reactive oxygen species; UV, ultraviolet; ECM, extracellular matrix; FDR, false discovery rate; SMC, smooth muscle cell.

**Table 3:** Summary of single-dose subcutaneous toxicity findings after DAP administration.

Endpoint	Control Group (0.9% Saline)		DAP Group (1.0 mL/head)	
	Male (n = 5)	Female (n = 5)	Male (n = 5)	Female (n = 5)
Mortality	0/5	0/5	0/5	0/5
Clinical signs	No abnormality	No abnormality	No abnormality	No abnormality
Body weight	No significant change ^a^	No significant change ^a^	No significant change ^a^	No significant change ^a^
Gross necropsy	No abnormality	No abnormality	No abnormality	No abnormality
Histopathology (injection site ^b^)	Minimal MNC infiltration (1/5, incidental ^c^)	No findings	No findings	No findings
ALD ^d^ (mL/head)	>1.0 mL/head (under the conditions of this study)			

^a^ Transient decreases in body weight were observed on day 2 in both groups, which recovered by day 4; no statistically significant differences were detected between the groups throughout the observation period (*p* > 0.05, Student’s *t*-test or Welch’s *t*-test). ^b^ The nape of the neck was selected as a standard site for subcutaneous administration in rodents. ^c^ Minimal mononuclear cell infiltration in the subcutis at the injection site in one male control animal (animal 1105) was judged to be an incidental finding caused by physical stimulation of the injection needle, with no toxicological significance. ^d^ ALD was determined based on the absence of mortality at the single dose tested (1.0 mL/head) under the conditions of this study; formal LD50 determination was not performed. Study conducted at HLB BioCode Co., Ltd. (study C26RA-075G) in compliance with Ministry of Food and Drug Safety Good Laboratory Practice regulations. DAP, dong-an pharmacopuncture; ALD, approximate lethal dose; MNC, mononuclear cell.

## Data Availability

The original contributions presented in this study are included in the article. Further inquiries can be directed to the corresponding author.

## Supplementary Materials

The following supporting information can be downloaded at https://www.mdpi.com/article/10.3390/ph19081317/s1, Table S1: Active compounds identified from the 11 constituent materials of DAP; Table S2: HPLC-UV assay results for asiaticoside in the DAP formulation used in this study; Table S3: Functional distribution of predicted dong-an pharmacopuncture targets according to component groups; Table S4: Representative GO and KEGG enrichment terms defining the four predicted mechanistic axes related to skin aging.

## Abbreviations

The following abbreviations are used in this manuscript:

ADME	Absorption, distribution, metabolism, and excretion
DAP	Dong-an pharmacopuncture.
DL	Drug-likeness
ECM	Extracellular matrix
FDR	False discovery rate
GLP	Good Laboratory Practice
H-C-T	Herb–compound–target
MMP	Matrix metalloproteinase
PPI	Protein–protein interaction
ROS	Reactive oxygen species
TCMSP	Traditional Chinese Medicine Systems Pharmacology
UV	ultraviolet
