# Supplementary material for: Mechanistic and Preliminary Safety Profiling of a Multicomponent Natural Product-Based Injectable Formulation Targeting Skin Aging-Related Pathways: A Network Pharmacology and Single-Dose Toxicity Study
Source: Pharmaceuticals (Basel). 2026 Aug 20;19(8):1317. doi: 10.3390/ph19081317 (PMC13516152; doi:10.3390/ph19081317)
Supplement: Supplementary file 1 [file pharmaceuticals-19-01317-s001.zip › pharmaceuticals-4461994-supplementary.pdf]

Supplementary Materials

Table S1. Active compounds identified from the 11 constituent materials of DAP.

Constituent material	Herb type	Active compound	No. of predicted targets	C–T evidence source
Moschus	Eight-component base group	Muscone	8	Literature
Fel Ursi	Eight-component base group	UDCA	17	STITCH + Literature
Fel Ursi	Eight-component base group	TUDCA	3	Literature
Calculus Bovis	Eight-component base group	Bilirubin	3	Literature
Calculus Bovis	Eight-component base group	Cholic acid	2	Literature
Calculus Bovis	Eight-component base group	Taurocholic acid	2	STITCH
Calculus Bovis	Eight-component base group	Glycocholic acid	2	STITCH
Calculus Bovis	Eight-component base group	Deoxycholic acid	0	Literature
Calculus Bovis	Eight-component base group	Glycocholate	1	STITCH
Calculus Bovis	Eight-component base group	Sodium deoxycholate	3	STITCH
Calculus Bovis	Eight-component base group	Taurodeoxycholate	1	STITCH
Scutellariae Radix	Eight-component base group	Baicalin	3	Literature
Scutellariae Radix	Eight-component base group	Baicalein	12	STITCH + Literature
Scutellariae Radix	Eight-component base group	Wogonin	3	STITCH
Scutellariae Radix	Eight-component base group	Quercetin	24	STITCH + Literature
Scutellariae Radix	Eight-component base group	Apigenin	8	STITCH
Scutellariae Radix	Eight-component base group	Chrysin	4	STITCH + Literature
Scutellariae Radix	Eight-component base group	Scutellarin	2	Literature
Scutellariae Radix	Eight-component base group	Oroxylin A	2	Literature
Scutellariae Radix	Eight-component base group	Isorhamnetin	6	STITCH + Literature
Scutellariae Radix	Eight-component base group	Kaempferol	6	STITCH
Scutellariae Radix	Eight-component base group	Scutellarein	1	STITCH
Scutellariae Radix	Eight-component base group	Acacetin	1	STITCH
Scutellariae Radix	Eight-component base group	Isoquercitrin	1	STITCH
Scutellariae Radix	Eight-component base group	Syringin	1	STITCH
Scutellariae Radix	Eight-component base group	4′-Dihydroxyflavone	1	STITCH
Phellodendri Cortex	Eight-component base group	Berberine	12	Literature
Phellodendri Cortex	Eight-component base group	Palmatine	3	Literature
Phellodendri Cortex	Eight-component base group	Obacunone	2	Literature
Phellodendri Cortex	Eight-component base group	Rutaecarpine	3	STITCH
Phellodendri Cortex	Eight-component base group	Limonin	1	STITCH
Pulsatillae Radix	Eight-component base group	Oleanolic acid	2	Literature
Pulsatillae Radix	Eight-component base group	Catechin	1	STITCH
Sophorae Tonkinensis Radix	Eight-component base group	Oxymatrine	2	Literature
Sophorae Tonkinensis Radix	Eight-component base group	Matrine	1	Literature

Sophorae Tonkinensis Radix	Eight-component base group	Formononetin	4	STITCH + Literature
Sophorae Tonkinensis Radix	Eight-component base group	Daidzein	5	STITCH
Sophorae Tonkinensis Radix	Eight-component base group	Genistein	15	STITCH
Sophorae Tonkinensis Radix	Eight-component base group	Emodin	9	STITCH + Literature
Sophorae Tonkinensis Radix	Eight-component base group	Kaempferol	6	STITCH
Aucklandiae Radix	Eight-component base group	Costunolide	2	Literature
Aucklandiae Radix	Eight-component base group	Dehydrocostus lactone	1	Literature
Aucklandiae Radix	Eight-component base group	Chlorogenic acid	1	Literature
P. quinquefolius	Three skin-oriented additional components	Ginsenoside Rb1	4	Literature
P. quinquefolius	Three skin-oriented additional components	Ginsenoside Rg1	1	Literature
P. quinquefolius	Three skin-oriented additional components	Ginsenoside Rg3	1	Literature
P. quinquefolius	Three skin-oriented additional components	Ginsenoside Rh2	1	Literature
P. quinquefolius	Three skin-oriented additional components	Ginsenoside Re	1	Literature
P. quinquefolius	Three skin-oriented additional components	Ginsenoside Rd	1	Literature
Hominis placenta	Three skin-oriented additional components	17-beta-Estradiol	6	STITCH + Literature
Hominis placenta	Three skin-oriented additional components	Estriol	2	STITCH
Hominis placenta	Three skin-oriented additional components	Estrone	2	STITCH
Hominis placenta	Three skin-oriented additional components	Progesterone	4	STITCH + Literature
Hominis placenta	Three skin-oriented additional components	Cortisone	3	STITCH + Literature
Hominis placenta	Three skin-oriented additional components	Hydrocortisone	2	STITCH + Literature
Hominis placenta	Three skin-oriented additional components	Aldosterone	4	STITCH + Literature
Hominis placenta	Three skin-oriented additional components	Melatonin	2	STITCH + Literature
Hominis placenta	Three skin-oriented additional components	Deoxycorticosterone	2	STITCH
C. asiatica	Three skin-oriented additional components	Asiaticoside	2	Literature
C. asiatica	Three skin-oriented additional components	Madecassoside	1	Literature
C. asiatica	Three skin-oriented additional components	Quercetin*	24	STITCH + Literature
C. asiatica	Three skin-oriented additional components	Apigenin*	8	STITCH

C. asiatica	Three skin-oriented additional components	Kaempferol*	6	STITCH
C. asiatica	Three skin-oriented additional components	Luteolin	1	STITCH
C. asiatica	Three skin-oriented additional components	Ursolic acid	3	Literature
C. asiatica	Three skin-oriented additional components	Chlorogenic acid*	1	Literature
C. asiatica	Three skin-oriented additional components	Ascorbic acid	1	Literature

*Compounds present in multiple constituent materials (quercetin: Scutellariae Radix and *C. asiatica*; apigenin: Scutellariae Radix and *C. asiatica*; kaempferol: Scutellariae Radix, Sophorae Tonkinensis Radix, and *C. asiatica*; chlorogenic acid: Aucklandiae Radix and *C. asiatica*). A total of 62 unique compounds were identified across 11 constituent materials.
C–T, compound–target; STITCH, Search Tool for Interactions of Chemicals (v5.0, combined confidence score ≥0.4); literature, compound–target interactions supported by published experimental evidence.

Table S2. HPLC-UV assay results for asiaticoside in the DAP formulation used in this study.

**Marker compound**	**Source component**	**Lot No.**	**Assay date**	**Detection method**	**Labeled content**	**Measured content**	**Specification**	**Judgment**
Asiaticoside	*C. asiatica*	NN0726001	2026-05-14	HPLC-UV, 206 nm	0.300 mg/vial	0.351 mg/vial	≥90% of labeled content	Pass

Asiaticoside was analyzed as a marker compound for *C. asiatica*. The measured values represented the reported assay results for the DAP batch used in this toxicity study. HPLC-UV, high-performance liquid chromatography–ultraviolet; DAP, dong-an pharmacopuncture.

Table S3. Functional distribution of predicted DAP targets according to component groups.

**Functional category**	**Eight-component base group**	**Three skin-oriented additional components**	**Overlapping targets between groups**	**Representative targets**
Hormone-related signaling	6	14	4	*ESR1*, *ESR2*, *AR*, *CYP19A1*, *PGR*, *SHBG*
Extracellular matrix/collagen remodeling	4	5	2	*COL1A1*, *TGFB1*, *MMP9*, *MMP13*, *AKT1*
Oxidative stress regulation	4	2	2	*NFE2L2*, *HMOX1*, *NOS3*, *BLVRA*
Inflammatory regulation	6	3	3	*TNF*, *IL6*, *PTGS2*, *CCL2*, *STAT1*, *MAPK8*
Senescence/longevity-related regulation	6	4	4	*SIRT1*, *TP53*, *FOXO3*, *CASP3*, *BAX*, *MCL1*
Melanogenesis-related regulation	1	2	0	*TYR*, *POMC*, *SLC23A2*

The eight-component base group refers to the constituent groups previously described in pharmacopuncture research. The three skin-oriented components are *P. quinquefolius*, Hominis Placenta, and *C. asiatica*. Target counts indicated the predicted or literature-supported targets assigned to each functional category. These data should be interpreted as a component group-level network summary and do not imply functional superiority, additive efficacy, or clinical effectiveness.

Table S4. Representative GO and KEGG enrichment terms defining the four predicted mechanistic axes related to skin aging.

Axis	Source	Term	Count	FDR	Fold enrichment
Inflammatory and oxidative stress-related regulation	KEGG	*TNF* signaling pathway	10	1.32×10^{-6}	11.76
Inflammatory and oxidative stress-related regulation	GO BP	Response to oxidative stress	9	1.99×10^{-5}	14.21
Inflammatory and oxidative stress-related regulation	KEGG	Chemical carcinogenesis–ROS	12	3.33×10^{-6}	7.40
Inflammatory and oxidative stress-related regulation	GO BP	Response to UV	4	1.38×10^{-2}	22.68
Hormonal skin homeostasis	KEGG	Steroid hormone biosynthesis	15	1.11×10^{-15}	31.80
Hormonal skin homeostasis	KEGG	Estrogen signaling pathway	10	3.44×10^{-6}	10.07
Hormonal skin homeostasis	GO MF	Steroid binding	14	2.99×10^{-22}	124.64
Hormonal skin homeostasis	GO MF	Estrogen response element binding	6	9.40×10^{-9}	150.54
Tissue repair and ECM remodeling	KEGG	*PI3K–Akt* signaling pathway	10	1.51×10^{-3}	3.87
Tissue repair and ECM remodeling	KEGG	*HIF-1* signaling pathway	7	2.72×10^{-4}	8.90
Tissue repair and ECM remodeling	GO BP	Negative regulation of apoptotic process	16	1.69×10^{-7}	8.19
Tissue repair and ECM remodeling	GO BP	Positive regulation of SMC proliferation	7	3.78×10^{-6}	38.14
Cellular senescence and longevity-related regulation	KEGG	Cellular senescence	8	2.63×10^{-4}	7.13
Cellular senescence and longevity-related regulation	KEGG	FoxO signaling pathway	9	1.74×10^{-5}	9.47
Cellular senescence and longevity-related regulation	KEGG	*AGE–RAGE* signaling pathway	14	8.68×10^{-12}	19.40
Additional molecular function terms	GO MF	Oxidoreductase activity	11	2.27×10^{-11}	37.48

Additional molecular function terms	GO MF	Nuclear steroid receptor activity	7	1.33×10^{-8}	74.30

Representative terms were selected from the full DAVID enrichment output based on FDR significance, gene count, fold enrichment, and biological relevance to skin aging-related mechanisms. The full enrichment output may be provided as an additional supplementary spreadsheet if required.
